# First clinical application of a novel duodenal mucosal ablation device for type 2 diabetes using radiofrequency vapor ablation

**DOI:** 10.1055/a-2344-7002

**Published:** 2024-07-03

**Authors:** Apostolis Papaefthymiou, Benjamin Norton, Pablo Becerra Hoebel, Leonardo Rodriguez Grunert, Rehan J. Haidry

**Affiliations:** 1 591481 Gastroenterology, Cleveland Clinic London, London, United Kingdom of Great Britain and Northern Ireland; 2 Clinica Colonial, Santiago, Chile

Duodenal mucosal ablation is an emerging endoscopic technique for the management of metabolic diseases, providing promising results for the control of type 2 diabetes 11 22 . The continuous advancement of endoscopic devices aims to optimize procedural efficacy, operability, and scalability 33 .

A novel through-the-scope ablation device, the circumferential radiofrequency vapor ablation system (Aqua Medical, Pleasanton, California, USA), is currently under evaluation in a first-in-human clinical trial (NCT05887635). This device consists of a 10.5-Fr through-the-scope catheter-based system with an ablation segment 2.5 cm in length ( Fig. 1Fig. 1 ). Energy is delivered to the duodenal mucosa in the form of heated water vapor generated within the catheter tip using radiofrequency. Positioning discs attached to the tip direct the distribution of vapor to the targeted duodenal tissue and allow circumferential mucosal ablation.

**Fig. 1:**
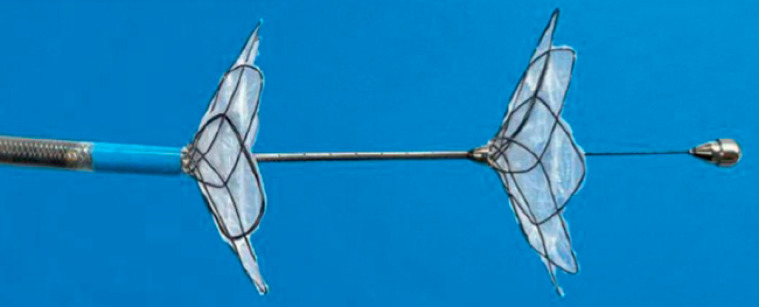
**Fig. 1** Circumferential radiofrequency vapor ablation (RFVA) system.

We present the case of a patient with poorly controlled type 2 diabetes who was eligible for recruitment and underwent the procedure under general anesthesia, using a double-channel endoscope ( Video 1Video 1 ). The patient was on metformin and had a fasting blood glucose level of 127 mg/dL and HbA1c of 8.3%. The duodenal mucosa was initially reviewed and washed with 2% N-acetylcysteine solution, and the fluid was suctioned. The ampulla was identified, representing the landmark of the proximal end of the area to be treated, and marked by deploying a clip on the contralateral wall. The first ablation was delivered immediately distal to the clip, and subsequent ablations were performed in a proximal-to-distal direction with minimal overlap. After the first series of ablations, the mucosa was reviewed, and a second series of ablations was carried out over the initial treatment zone ( Fig. 2Fig. 2 ). Finally, the entire ablated segment was reviewed for complications and remaining nonablated segments. The patient was monitored for 24 hours post-procedure and remained asymptomatic. No adverse events were recorded prior to discharge. Follow-up after 1 month confirmed the absence of adverse events and a reduction in fasting blood glucose (118 mg/dl) and HbA1c (6.5%) levels.

Demonstration of the use of the novel duodenal mucosal ablation device using radiofrequency vapor ablation (RFVA) in a patient with type 2 diabetes.Video 1Video 1

**Fig. 2:**
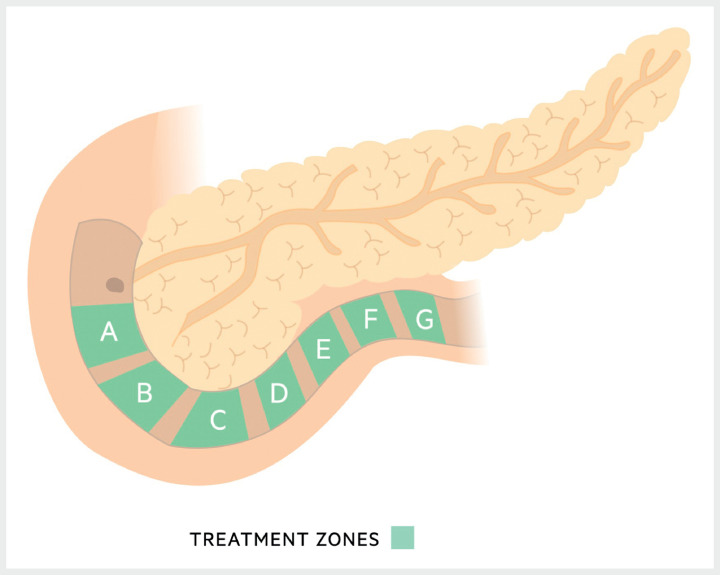
**Fig. 2** Treatment zones in the duodenum for duodenal mucosal ablation.

This new circumferential radiofrequency vapor ablation system for duodenal mucosal ablation presents a promising intervention for type 2 diabetes. Results from the first-in-human STEAM T-2DM trial will provide insights into the overall safety and efficacy of this technique.

Endoscopy_UCTN_Code_TTT_1AO_2AN

## Notes

Endoscopy E-Videos https://eref.thieme.de/e-videos
*E-Videos* is an open access online section of the journal *Endoscopy* , reporting on interesting cases and new techniques in gastroenterological endoscopy. All papers include a high-quality video and are published with a Creative Commons CC-BY license. Endoscopy E-Videos qualify for HINARI discounts and waivers and eligibility is automatically checked during the submission process. We grant 100% waivers to articles whose corresponding authors are based in Group A countries and 50% waivers to those who are based in Group B countries as classified by Research4Life (see: https://www.research4life.org/access/eligibility/ ). This section has its own submission website at https://mc.manuscriptcentral.com/e-videos .
